# Raman and Luminescent Spectra of Sulfonated Zn Phthalocyanine Enhanced by Gold Nanoparticles

**DOI:** 10.1186/s11671-017-1972-5

**Published:** 2017-03-16

**Authors:** V. Kavelin, O. Fesenko, H. Dubyna, C. Vidal, T. A. Klar, C. Hrelescu, L. Dolgov

**Affiliations:** 1 Institute of Physics of NAS of Ukraine, 46, Nauky Ave, Kyiv, 03680 Ukraine; 2 Institute of Applied Physics, Johannes Kepler University Linz, Linz, 4040 Austria; 3 Institute of Physics, University of Tartu, 1, Ostwaldi, Tartu, 50411 Estonia

**Keywords:** Sulfonated Zn phthalocyanine (ZnPC_Sulf_), Au-SiO_2_ nanoparticles, Surface-enhanced Raman spectroscopy (SERS), Surface-enhanced infrared absorption (SEIRA)

## Abstract

Sulfonated Zn phthalocyanine, as a prospective photosensitizer in the photodynamic therapy of tumors, is investigated by means of Raman, infrared, and fluorescence spectroscopies. Conventional and surface-enhanced spectra from this photosensitizer are obtained and compared. Gold nano-islands attached to silica cores (Au-SiO_2_) are proposed as nanostructures providing plasmonically enhanced signals. Pronounced enhancement of Raman and infrared spectral bands from sulfonated Zn phthalocyanine allows their more convenient assignment with vibrational modes of sulfonated Zn phthalocyanine. In comparison to Raman and IR, the fluorescence is less enhanced by Au-SiO_2_ particles.

## Background

The application of phthalocyanine compounds, in organic solar cells [1] or for cancer treatment [2, 3], renewed the research interest in such compounds, initially used only as dyes. Sulfonated metastable α-zinc phthalocyanine (α-ZnPc) and the stable β-zinc phthalocyanine (β-ZnPc) are not soluble in water [4]. Water solubility can be achieved by modifying the 3-sulfate-substituted ZnPc with L-cysteine radicals.

Noble metal nanoparticles can be used as optical nanoantennas for the enhancement of visible and infrared spectral signals for the detection of minute amount of analytes [5, 6]. Here, we propose specially prepared hybrid nanoparticles (Au-SiO_2_) for surface-enhanced Raman (SERS), surface-enhanced infrared (SEIRA), and visible range spectroscopies of phthalocyanine-based molecules. Our hybrid nanoparticles consist of dielectric silica nanospheres with diameters of 180 nm decorated with gold nano-islands with diameters in the range of 10–30 nm. In our case, gold nano-islands are randomly interconnected, in contrast to other reports where the dielectric cores were covered by a continuous shell [6–8]. Such incomplete, but dense, coverage of the dielectric cores with nano-islands is prone to enhance the Raman signals more than a complete homogeneous gold shell [9]. Here, we demonstrate that Au-SiO_2_ nanoparticles with an incomplete gold shell morphology can serve as SERS-SEIRA substrates for the detection of ZnPC_Sulf_ in the visible and infrared spectral range, while in earlier reports, only gold electrodes [10] or silver islands [11] were used for SERS detection of ZnPC_Sulf_.

Both plasmonic enhancement and charge transfer from the nanoparticles to the ZnPC_Sulf_ molecules through the specific covalent thiol-gold (S-Au) bonds can be considered as possible enhancement mechanisms for surface-enhanced spectroscopy. Since in our case, the spectral ranges at which the SERS effect caused by charge transfer and plasmonic resonance are spectrally overlapped and are hardly separable; we associate the measured SERS enhancements of ZnPc signals with both mechanisms. Impact of chemical enhancement, namely possible charge transfer from the substrate to the analyte molecules, can be investigated separately on the examples of analytes deposited on graphene surface [12]. The SERS/SEIRA enhancements allow a better than for conventional spectroscopy assignment of observed spectral bands with molecular vibrations, rendering our Au-SiO_2_ nanoparticles as promising SERS-SEIRA substrates with satisfactory enhancement of broadband spectral signals.

## Methods

**Fig. 1:**
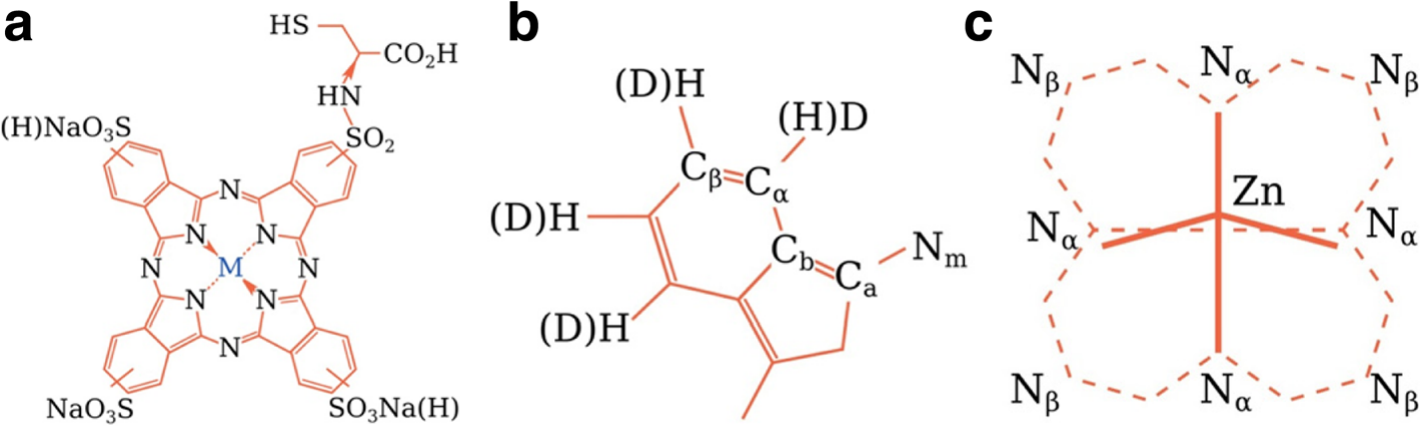
Schematic representation of ZnPC_Sulf_ molecule and designation of standard connections. **a** ZnPC_Sulf_ molecule. **b** Isoindole radical. **c** Pyrrole group or macrocycle

Both 3-sulfate-substituted ZnPc and ZnPc with one substituent modified by L-cysteine radicals were produced by the NIOPIK company (Russia) with a purity of 92%. Here, only the main steps of synthesis are noted. Particularly, trisodium salt of zinc phthalocyanine was synthesized from trisulfonic acid by sulfonation of unsubstituted phthalocyanine with chlorosulfonic acid in inert high-boiling solvents (o-dichlorobenzene, trichlorobenzeze) [13]. The schematic representation of ZnPC_Sulf_ molecule and designation of standard connections are shown in Fig. 1. The replacement of one of the sulfate functional groups of the ZnPC_Sulf_ by L-cysteine was conducted according to ref. [14].

Monodispersed 150–180 nm silica spheres were prepared by the Stöber method from tetraethyl orthosilicate as a dispersion in ethanol [15]. Gold seeds of 5–10 nm in diameter were prepared in water by reduction of chloroauric acid (HAuCl_4_) with tetrakis(hydroxymethyl)phosphonium chloride working also as a stabilizing agent. Silica nanoparticles were functionalized with (3-amino)propyltrimethoxysilane. When silica and gold nanoparticle dispersions are mixed, the resultant terminal amine groups on the silica surface act as attachment points for the small gold seeds [8]. After washing by centrifugation, the hybrid nanoparticles (Au-SiO_2_) were finally redispersed in water (~10^9^ 1/cm^3^) by ultrasonication.

A solution of 10 μg/ml of ZnPC_Sulf_ in water was used as reference. Then, 100 μl of this solution was mixed with 20 μl of Au-SiO_2_ NP dispersion (concentration of NPs ~10^9^ 1/ml). These tested and reference mixtures were used without additional modifications in fluorescence and light extinction measurements. The samples for Raman and infrared measurements were prepared in a similar way. Namely, equal amounts of 20 μl droplets from tested and reference mixtures were drop-casted on a glass (for Raman) and on BaF_2_ substrates (for infrared) and dried at room temperature. Line scans of the droplets from tested and reference mixtures were compared in terms of their Raman and infrared spectra.

The Raman spectra were obtained with an inVia micro-Raman spectrometer (Renishaw plc, Wotton-under-Edge, UK) at the HeNe laser excitation wavelength of 633 nm. IR spectra were measured by FTIR spectrometer (VERTEX, Bruker, Germany). Light extinction by the solutions was measured by the Cary 500 Scan UV-Vis-NIR Spectrometer. Luminescence of solutions was measured by FluoroLog-2 spectrofluorimeter (Instruments SA). All measurements were performed at room temperature.

Raman and IR experiments probing intrinsic fingerprint spectra of analytes were carried out in dry environment because they are often used in the format of drop casting and drying. In contrast, fluorescence enhancement assays need a detection step in aqueous environment, and hence, we measured fluorescence in aqueous solution [16].

## Results and Discussion

### Optical Properties of Au-SiO_2_ Nanoparticles

**Fig. 2:**
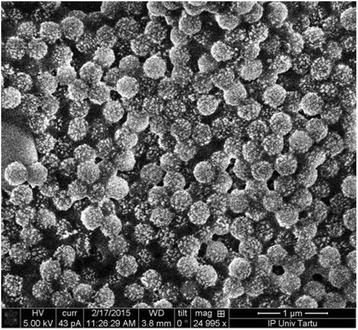
Scanning electron microscopy of precipitated Au-SiO_2_ nanoparticles

The hybrid Au-SiO_2_ nanoparticles consist of dielectric silica nanospheres with diameters of 180 nm decorated with gold nano-islands with diameters in the range of 10–30 nm. In Fig. 2, a scanning electron microscopy (SEM) image of representative Au-SiO_2_ nanoparticles is shown. The Au-SiO_2_ nanoparticles possess a corrugated surface caused by separated as well as partially connected gold nano-islands.

**Fig. 3:**
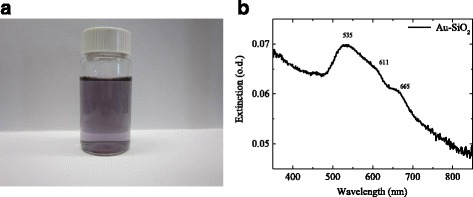
**a** Water dispersion of Au-SiO_2_ nanoparticles. **b** Optical density (OD) of an aqueous dispersion of Au-SiO_2_ nanoparticles in a cuvette with 10 mm of optical path length, corrected by the OD of a cuvette filled with water without nanoparticles

Solutions of Au-SiO_2_ nanoparticles dispersed in water exhibit a blue-violet color (Fig. 3a).

The extinction of the Au-SiO_2_ nanoparticles, as shown in Fig. 3b, can be explained by the Mie light scattering from the SiO_2_ particles together with a plasmon peak at 535 nm which is expected for gold nanoparticles with dimensions in the range of 10–30 nm [17]. The additional spectral shoulder at 611 and 665 nm is most probably caused by plasmonic coupling of neighboring gold nano-islands on the silica cores.

**Fig. 4:**
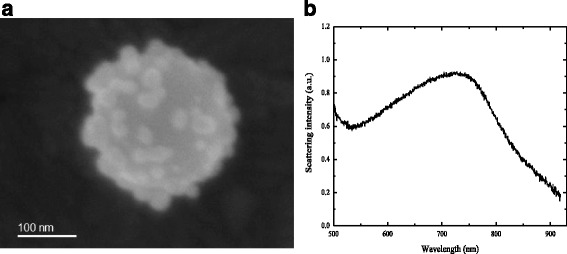
**a** SEM image of a representative Au-SiO_2_ nanoparticle. **b** Scattering spectrum of the Au-SiO_2_ nanoparticle depicted in (**a**)

The combination of SEM imaging with subsequent dark field spectroscopy of the same single nanoparticles allowed us to measure the light-scattering spectrum from a selected individual Au-SiO_2_ nanoparticle (Fig. 4a) and to correlate its shape and size directly to its scattering spectrum (Fig. 4b). This particular example shows a broad maximum at 750 nm. The maximum at 750 nm can be attributed to the coupling of several plasmon resonances from the gold nano-islands attached to the silica core.

### Extinction Spectra of ZnPC_Sulf_ Solutions With and Without Au-SiO_2_

**Fig. 5:**
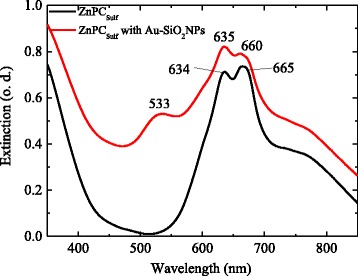
UV-VIS spectra of ZnPC_Sulf_ (*black line*) and ZnPC_Sulf_ with Au-SiO_2_ nanoparticles (*red line*)

The extinction spectrum of ZnPC_Sulf_ aqueous solutions with and without Au-SiO_2_ is shown in Fig. 5. The increased extinction between 350 and 450 can be attributed to the Soret band (B band) absorption. The two maxima at 634 and 665 nm can be attributed to the main absorption within the Q band of ZnPC_Sulf_ [18, 19].

The addition of Au-SiO_2_ causes the appearance of additional maximum at 533 nm associated with the plasmon resonances of the gold nano-islands. Further, the Mie light scattering from the silica cores causes an overall increase and reshaping of the extinction spectra of ZnPC_Sulf_ solutions with Au-SiO_2_ compared to the extinction spectra of pure ZnPC_Sulf_ solutions.

### Luminescence of ZnPC_Sulf_ Solutions With and Without Au-SiO_2_

**Fig. 6:**
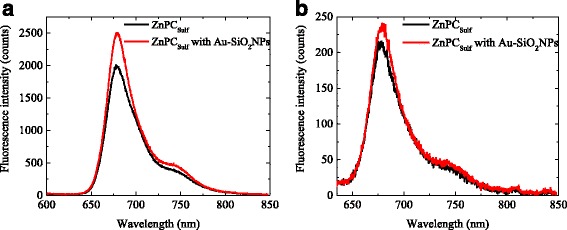
Fluorescence spectra of ZnPC_Sulf_ solutions with and without Au-SiO_2_ nanoparticles: **a** for an excitation resonant to the Soret band of the ZnPC_Sulf_ (*λ*_exc_ = 405 nm) and (**b**) for an excitation wavelength resonant to the dipolar plasmon mode of the gold nano-islands (*λ*_exc_ = 532 nm)

The luminescence spectra from ZnPC_Sulf_ solutions exhibit a main band at 675 nm and a shoulder at 750 nm. The spectral positions of these emission bands do not change for different excitation wavelengths, *λ*_exc_ = 405 and 532 nm, Figs. 6a, b, respectively. The fluorescence is more pronounced for *λ*_exc_ = 405 nm because of a higher absorption coefficient of ZnPC_Sulf_ at 405 nm (Soret band) than that at 532 nm. The fluorescence emission coincides with the electronic HOMO-LUMO (Q bands) transitions [20].

When the ZnPC_Sulf_ solutions containing Au-SiO_2_ nanoparticles are excited within the Soret band of the ZnPC_Sulf_ (excitation wavelength at *λ*_exc_ = 405 nm in Fig. 6a), the detected fluorescence intensity is more than 20% higher compared to the intensity of the ZnPC_Sulf_ solutions without Au-SiO_2_ nanoparticles.

The distance between the metal nanoparticles and fluorophores and the orientation of the molecular dipoles determine if the fluorescence is enhanced or quenched [19]. If the position of the fluorophores is at a distance smaller than 5 nm from the metal nanoparticle, the fluorescence may be quenched by energy transfer. In addition, quenching can be achieved by out-of-phase dipole coupling. Energy transfer acts on short range (10 nm and less); out-of-phase effects act on ranges up to 20 nm [21].

If, however, the molecular dipole and the image dipole act in phase, an enhancement of fluorescence can be expected at distances of tens of nanometers [22, 23]. At such distances, the quenching is less probable but the fluorophores are still situated in proximity of the nanoparticles. Additionally, locally enhanced electric fields lead to a higher excitation probability close to the metal nanoparticles. In total, if the fluorescent dipole has a proper orientation with respect to the nanoparticle surface, the excitation and the radiative rate of the fluorophores are increased. Even in these conditions, experimental observation of plasmon-coupled fluorescence is not easy for single particles.

Since in solution, the distance between ZnPC_Sulf_ molecules and Au-SiO_2_ nanoparticles depends on concentration and since the nanoparticles and fluorophore molecules move stochastically in solution, no accurate control of the distance between the ZnPC_Sulf_ molecules and Au-SiO_2_ nanoparticles was achieved. However, in both experiments, no quenching but fluorescence enhancement was observed (Table 1). The fact that excitation of the ZnPC_Sulf_ solutions with Au-SiO_2_ nanoparticles, resonant to the Soret band of the ZnPC_Sulf_, leads to a more pronounced fluorescence enhancement (of 20%) with respect to the reference than the excitation resonant to the dipolar mode of the gold nano-islands can be carefully attributed to the higher Mie scattering of the SiO_2_ nanoparticles at 405 nm than that at 532 nm, which might cause a higher absorption probability. It seems that the local field enhancement occurring due to resonant excitation of the dipolar plasmon mode of the gold nano-islands does not lead to a substantial increase in the excitation probability of the ZnPC_Sulf_ molecules. More pronounced fluorescence enhancements might be obtained from aggregates of several metal nanoparticles, where a proper spacing of the fluorophores to the metal is achieved and where several strong hotspots occur in the gaps between the nanoparticles [24].

**Table 1:** Luminescence of ZnPC_Sulf_ with and without Au-SiO_2_ nanoparticles for different excitation wavelengths

Excitation wavelength	Emission wavelength, nm	Intensity		Relative enhancement %
		ZnPC_Sulf_	ZnPC_Sulf_ with Au-SiO_2_	
405 nm (Soret band)	679.5	2000	2550	26
	742	399	497	20
532 nm (resonant to the gold nano-island plasmons)	680.6	216	242	12
	742	45	50	9

### IR Spectra of ZnPC_Sulf_ Samples

**Fig. 7:**
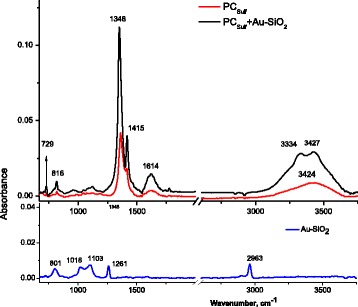
IR spectra of ZnPC_Sulf_, ZnPC_Sulf_ with Au-SiO_2_, and pure Au-SiO_2_

The IR spectra of ZnPC_Sulf_ samples with and without Au-SiO_2_ as well as the band assignments of the detected spectral bands with the bond vibrations in the Zn phthalocyanine molecules are shown in Fig. 7. Surface-enhanced infrared absorption (SEIRA) for ZnPC_Sulf_ containing Au-SiO_2_ was detected. The infrared bands of ZnPC_Sulf_ were enhanced up to five times by the Au-SiO_2_ depending on the type of molecular group. Commonly in SEIRA, this enhancement is provided by electromagnetic interactions due to the surface plasmon resonance in the metal nanostructures and by changes in molecular dipole moments when the molecules are adsorbed on metal nanostructures [25]. In our case, the dominant role in the enhancement could be played by the molecular mechanism due to the fact that the vibronic (IR spectrum) of ZnPC_Sulf_ overlaps only partially with the spectral tail of the plasmon resonances of the Au-SiO_2_ nanoparticles and hence, the electromagnetic enhancement is not high.

The IR spectra of ZnPC_Sulf_ exhibit a band at 1614 cm^−1^, which is characteristic for compounds containing the benzene rings and connected with C-C stretching vibrations (Fig. 7) [26]. The shift of this band to lower frequencies by 5 cm^−1^ can be caused by deuteration.

The bands at frequencies 1415 and 1348 cm^−1^ are related to isoindole stretching and coupling of pyrrole and isoindole stretching vibrations, respectively [27]. The frequencies of in-plane vibrations with large contributions of the C-C-H mode fall within the 1000–1300 cm^−1^ range. Two bands at frequencies 729 and 816 cm^−1^ relate to in-plane skeletal vibrations (Table 2) [26].

**Table 2:** Assignment of the IR spectral bands with molecular vibrations in ZnPC_Sulf_

ZnPC_Sulf_, *v* (cm^−1^)	ZnPC_Sulf_ with AuSiO_2_, *v* (cm^−1^)	Assignment
3424	3427	C-C stretching vibrations of pyrrole ring, O-H, N-H
1619	1614	C-C stretching vibrations of the benzene rings, C = C
1409	1415	Isoindole stretching coupling of pyrrole, C-H
1354	1348	C-C-H
806	816	C-H deformations of the isoindole ring, plane skeletal vibrations
737	729	Plane skeletal vibrations

### Raman Spectra of ZnPC_Sulf_ Samples

**Fig. 8:**
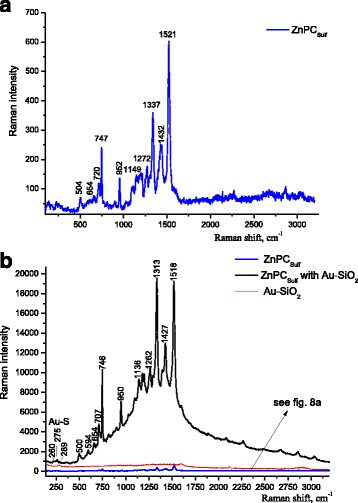
Raman spectra of ZnPC_Sulf_ (**a**), ZnPC_Sulf_ mixed with Au-SiO_2_ nanoparticles, and pure Au-SiO_2_ nanoparticles (**b**)

Raman spectra of ZnPC_Sulf_ are shown in Fig. 8a. Most of the ZnPC_Sulf_ Raman bands correspond to deformations and stretching vibrations of chemical bonds of carbon atom with hydrogen, nitrogen, and characteristic vibrations of nitrogen-zinc bonds (Fig. 8). It is known that the most intense Raman peaks appeared from the non-polar functional groups, due to stronger change of dipole moments and their polarizability [28]. Therefore, the oscillations of double and triple carbon bonds and aromatic groups of symmetric vibrations are significantly enhanced in comparison with such as C-H; O-H; C = O; S-H [29].

For the range of low frequencies (~1000 to 100 cm^−1^), it is typical to observe intra-molecular and crystal vibrations [28]. Since the ZnPC_Sulf_ molecule does not have hydrogen atoms that directly connect with the ring surrounding of the central metal atom, the stretching vibrations of C-H bonds have a much higher frequency compared to the frequency of the macrocycle fluctuations. The C-H vibrations should appear around 3000 cm^−1^ and have a low intensity [30].

Symmetric valence and deformation vibrations of Zn-N_a_ bonds [31] are connected with the peak at 747 cm^−1^ (Fig. 8a). The most intense peak located at 1521 cm^−1^ (Fig. 8a) can be attributed to vibrations of completely symmetrical bonds, such as symmetric valence C_a_ = C_в_ vibrations in benzene rings and C_a_ = N_в_ vibrations in pyrrole structures [32]. In the spectral range below 1000 cm^−1^, vibrational motion of the pyrrole groups dominates in the spectra.

Mixing of ZnPC_Sulf_ with Au-SiO_2_ nanoparticles resulted in the significant enhancement of its Raman signal (Fig. 8b).

As a result of ZnPC_Sulf_ interaction with gold, the peaks at 1521 and 1337 cm^−1^ are shifted and significantly increased. The increase of intensity can be attributed to pyrrole molecules chemically bonded with gold [10]. The peak at 952 cm^−1^ corresponds to out-of-plane vibrations of C-H bonds. Similar vibrations occur in the bis- and three-phthalocyanine structures.

The changes in intensity and position of Raman spectral bands caused by addition of Au-SiO_2_ nanoparticles to the ZnPC_Sulf_ are depicted in Table 3.

**Table 3:** Assignment of the Raman spectral bands with molecular vibrations in the ZnPC_Sulf_

ZnPC_Sulf_, cm^−1^	ZnPC_Sulf_with Au-SiO_2_, cm^−1^	Assignment	Reference	I_ZnPCSulf with Au-SiO2_ /I_ZnPCSulf_
146	145	Zn-N, pyrrole out-of-plane, N_a_-C_a_-N_b_	[32]	20
–	260	radial Au-S stretching modes	[33]	–
–	275	Au-S vibration	[33]	–
–	289	Au-S vibration	[33]	–
504	500	Macrocycle bending of pyrrole	[31]	25
594	594	Out-of-plane C-H, C-N-C, deformations of the isoindole ring	[32]	30
654	654	C-C-C benzene, C-S	[31]	36
720	707	C_a_, N_a_, out-of-plane, C-S	[31]	40
747	746	C_a_-N-C_a_, C-C-N, Zn-N_a_, antisymmetric deformation of the macrocycle	[32]	42
952	950	C-H out-of-plane, C-C-C pyrrole, and vibrations of benzene groups	[34]	50
1149	1136	C_a_-C_б_, C-H benzene, C_б_-C_б_, stretching vibrations of pyrrole groups	[34]	65
1272	1262	C_a_-N, N-C_a_-N_a_, C_a_-N-C_a_	[31]	60
1337	1313	H-C_a_-C_b_, C_a_ = N_b_, C_a_-C_b_,	[32]	56
1432	1427	C_a_ = C_b_	[32]	51
1521	1518	C_a_ = C_b_, C_a_ = N_b_, and stretching vibration in the pyrrole group are totally symmetric vibration	[31]	32

As one can see from Table 3, there are changes in the position of ZnPC_Sulf_ spectral bands caused by Au-SiO_2_ nanoparticles. These changes are connected with fluctuations of bonds in pyrrole ring and can be represented as 720 cm^−1^ → 707 cm^−1^, 1149 cm^−1^ → 1136 cm^−1^, 1272 cm^−1^ → 1262 cm^−1^, and 1337 cm^−1^ → 1313 cm^−1^. Particularly, the peaks at 1337 and 1521 cm^−1^ correspond to the valence and deformation vibrations of pyrrole rings. Reduction-oxidation processes in ZnPC_Sulf_ can be located at the ligand and at the metal center of ZnPC_Sulf_. The ZnPC_Sulf_ ring can undergo successive one-electron reduction and one-electron oxidation to yield the anion and cation radicals.

The thiol group (H-S) reacts with Au nanoparticles and forms very stable covalent metal-sulfur bonds [33]. Usually, the characteristic peak for the S-H group of ZnPC_Sulf_ is observed at 2546 cm^−1^, but in case of ZnPC_Sulf_ mixed with Au-SiO_2_ nanoparticles, no such specific band was detected. The absence of the S-H peak indicates that there was a chemical interaction with the formation of S-Au bond [34]. As an important feature in the spectra of ZnPC_Sulf_ with Au-SiO_2_, multiple Au-S stretching modes were observed at about 250 cm^−1^ (black spectra in Figs. 8b). Nanostructures, such as Au-SiO_2_ nanoparticles, should give rise to multiple Au-S stretching modes at different frequencies due to the different nature of the Au-S bonds involved (Au-S within the staple structure, where ZnPC_Sulf_ can be confined between different gold islands or different Au-SiO_2_ nanoparticles and Au-S involving a core gold atom) [35].

The enhancement factor for spectral signals of some molecular groups of ZnPc reaches up 65 times for SERS and 5–6 times in SEIRA effect. This can be deduced by the fact that a molecular mechanism is only present in IR spectroscopy because SEIRA experiments were carried out on the tail of the plasmon resonance. However, chemical and electromagnetic mechanisms of enhancement play a role in Raman spectroscopy because a laser with a wavelength of 633 nm is used, which almost coincides with one of the plasmonic frequencies which excited in the gold islands (see Fig. 3b). The appearance of Au-S bands at 275 and 289 cm^−1^ points on a chemical bond between Au and ZnPc.

## Conclusions

Conventional and surface-enhanced spectra from 3-sulfate-substituted zinc phthalocyanine having the L-cysteine radicals are investigated and compared. Plasmonic gold nano-islands attached to silica cores showed potential as universal SEIRA-SERS broadband substrates. These nanostructures are suitable both for a moderate enhancement of fluorescence and for essential enhancements of Raman and IR signals of sulfonated Zn phthalocyanine. The enhancement of observed Raman signal is mainly caused by plasmonic mechanism of SERS. The chemical contribution to enhancement is possibly caused by charge transfer through the Au-S bonds, according to the Raman spectral data. The SERS spectral bands show significant enhancement and some shifts of spectral peaks when compared with ordinary Raman scattering spectra. At the same time, the plasmonic influence on the fluorescence of sulfonated Zn phthalocyanine appeared moderate, which can be attributed to the random spacing between the ZnPC molecules and Au-SiO_2_ nanoparticles. The weaker, as compared to the Raman, enhancement can also be explained by the fact that in case of fluorescence, we measured ensembles of nanoparticles in solution while, in case of Raman and IR measurements, the signals were obtained from dense layers of closely packed particles.

Au-SiO_2_ nanoparticles are promising candidates as SERS-SEIRA substrates for broadband spectroscopic material identification of very thin films and monolayers of biological molecules. The presented results can be useful for the elaboration of SEIRA and SERS sensors for detection of small amounts of chemical reagents and their spectral characterization.

## Abbreviations

Au-SiO_2_: Silica core-gold shell nanoparticle
HOMO: Highest occupied molecular orbital
IR: Infrared spectroscopy
LUMO: Lowest unoccupied molecular orbital
SEIRA: Surface-enhanced infrared absorption
SEM: Scanning electron microscope
SERS: Surface-enhanced Raman spectroscopy
ZnPc: Zn phthalocyanine
ZnPC_Sulf_: Sulfonated Zn phthalocyanine
